# TAM receptor ligands in lupus: Protein S but not Gas6 levels reflect disease activity in systemic lupus erythematosus

**DOI:** 10.1186/ar3088

**Published:** 2010-07-16

**Authors:** Chang-Hee Suh, Brendan Hilliard, Sophia Li, Joan T Merrill, Philip L Cohen

**Affiliations:** 1 Section of Rheumatology, Department of Medicine, Temple University School of Medicine, 3322 North Broad Street, Room 205, Philadelphia, PA 19140, USA; 2 Department of Allergy-Rheumatology, Ajou University School of Medicine, Woncheon-dong San 5, Youngtong-gu, Suwon 443-721, Korea; 3 Clinical Pharmacology Research Program, Oklahoma Medical Research Foundation, 825 N.W. 13th Street, Oklahoma City, OK 73106, USA

## Abstract

**Introduction:**

The TAM (tyro 3, axl, mer) kinases are key regulators of innate immunity and are important in the phagocytosis of apoptotic cells. Gas6 and protein S are ligands for these TAM kinases and bind to phosphatidyl serine residues exposed during apoptosis. In animal models, absence of TAM kinases is associated with lupus-like disease. To test whether human systemic lupus erythematosus (SLE) patients might have deficient levels of TAM ligands, we measured Gas 6 and protein S levels in SLE.

**Methods:**

107 SLE patients were recruited. Of these, 45 SLE patients were matched age, gender and ethnicity with normal controls (NC). Gas6 and free protein S were measured with sandwich enzyme linked immunosorbent assays (ELISAs).

**Results:**

Overall, the plasma concentrations of Gas6 and free protein S were not different between 45 SLE patients and 45 NC. In SLE patients, the levels of free protein S were positively correlated with age (r = 0.2405, *P* = 0.0126), however those of Gas6 were not. There was no correlation between the concentrations of Gas6 and free protein S in individuals. Levels of free protein S were significantly lower in SLE patients with a history of serositis, neurologic disorder, hematologic disorder and immunologic disorder. Gas6 levels were elevated in patients with a history of neurologic disorder. The SLE patients with anti-Sm or anti-cardiolipin IgG showed lower free protein S levels. Circulating free protein S was positively correlated with complement component 3 (C3) (r = 0.3858, *P* < 0.0001) and complement component 4 (C4) (r = 0.4275, *P* < 0.0001). In the patients with active BILAG hematologic involvement, the levels of free protein S were lower and those of Gas6 were higher.

**Conclusions:**

In SLE, free protein S was decreased in patients with certain types of clinical history and disease activity. Levels of free protein S were strongly correlated with C3 and C4 levels. Gas6 levels in SLE patients differed little from levels in NC, but they were elevated in the small numbers of patients with a history of neurological disease. The correlation of decreased protein S levels with lupus disease activity is consistent with a role for the TAM receptors in scavenging apoptotic cells and controlling inflammation. Protein S appears more important functionally in SLE patients than Gas6 in this regard.

## Introduction

Systemic lupus erythematosus (SLE) is a chronic autoimmune disease with diverse presentations. Its pathogenesis remains elusive; however, multifactorial interactions among genetic and environmental factors may be involved [1,2]. SLE is characterized by dysregulation of the immune system that involves hyperactivity of T cells and B cells, production of pathogenic autoantibodies, and the formation of immune complexes, which can lead to multiorgan damage.

Certain nuclear and cytoplasmic autoantigens become clustered in the surface blebs of apoptotic cells [3]. Under normal circumstances, apoptotic cells are engulfed by macrophages in the early phase of cell death without inducing inflammation or the immune response. In SLE, however, the clearance of apoptotic cells by macrophages is impaired, which may allow apoptotic cells to serve as immunogens for the induction of autoreactive T and B cells and drive the production of autoantibodies [4].

The reasons for the defective clearance of apoptotic cells in SLE are not clear. The past decade has provided significant evidence that complement deficiencies, immunoglobulin (Ig) M deficiency, pentraxin deficiency and defects in macrophage handling may each contribute to defective clearance of apoptotic bodies [5-7]. Macrophages recognize apoptotic cells through an array of surface receptors. Among them, the tyro 3, axl, mer (TAM) kinases, especially the c-mer receptor tyrosine kinase, play an especially important role in the clearance of apoptotic cells [8,9]. Mice lacking c-mer have impaired clearance of apoptotic cells and develop progressive lupus-like autoimmunity [10]. The two ligands that bind to and activate c-mer are growth arrest-specific 6 (Gas6) and protein S, which in turn bind to phosphatidylserine residues exposed early in apoptosis on the surface of the apoptotic cell [11-14].

Gas6, a 75 kDa multimodular vitamin K-dependent protein that has 46 to 48% amino acid identity to protein S, was discovered in the early 1990 s [15]. It contains an N-terminal γ-carboxyglutamic acid (Gla) domain, interacting with phosphatidylserine containing membranes, followed by four epidermal growth factor-like domains and a large C-terminal region homologous to the sex hormone binding globulin, can ligate TAM receptor tyrosine kinases [16]. Gas6 is expressed in many tissues, including capillary endothelial cells, vascular smooth muscle cells, and bone marrow cells. Unlike protein S, Gas6 is not expressed in the liver, and its concentration in plasma is 1,000-fold lower than that of protein S [17].

Protein S has a critical function in regulating coagulation by serving as a cofactor for activated protein C-dependent proteolytic inactivation of factor Va and factor-VIIIa. Protein S circulates as approximately 40% free protein S and 60% as a complex with C4-binding protein; only free protein S is active as a cofactor for activated protein C and a ligand for the TAM receptor kinases. In the absence of free protein S, there is increased risk of thromboembolism [18].

It is reasonable to hypothesize that Gas6 and protein S might have important roles in the pathogenesis of SLE. Recently, plasma Gas6 was reported to be elevated in patients with severe sepsis, septic shock, and severe acute pancreatitis [19-21]. However, there are no reports about Gas6 levels in SLE. Low levels of protein S are reported in SLE, and could be contributing to the thrombotic propensity in certain SLE patients [22-24]. We have therefore compared Gas6 and free protein S concentrations in patients with SLE, examining their possible use as biomarkers of clinical phenotype and/or disease activity.

## Materials and methods

### Subjects

Samples from 107 SLE patients, participating in the Oklahoma Cohort for Rheumatic Disease, were studied. All patients satisfied at least four of the 1982 revised American College of Rheumatology (ACR) criteria for SLE [25]. Forty-five of these SLE patients were matched by age, gender and ethnicity to healthy normal controls (NC) (Table 1). Heparinized plasma samples were collected and stored at -70°C immediately after collection. Information on medical history, ACR criteria for SLE, and current disease activity was registered into a database, which included no personal identifiers. Laboratory data included blood cell counts, routine chemistry, urinalysis, complement levels, anti-dsDNA, anti-Sm, anti-RNP, anticardiolipin (ACA) IgG and IgM, lupus anticoagulant (LAC), anti-β2 glycoprotein I, anti-Ro, anti-La, and anti-protein S antibody. C3 and C4 were measured in the Oklahoma Medical Research Foundation clinical laboratory by standard nephelometric techniques. Disease activity was scored using the Systemic Lupus Erythematosus Disease Activity Index (SLEDAI) and the British Isles Lupus Assessment Group (BILAG) Instrument [26,27].

**Table 1:** Characteristics of patients

	Lupus matched (*n *= 45)	Normal control (*n *= 45)	Lupus unmatched (*n *= 62)
Age (years)	40.47 ± 15.5	41.38 ± 15.9	37.27 ± 13.02
Sex (F:M)	34:11	34:11	51:11
Ethnicity			
Caucasian	41	41	54
African	2	2	2
Asian	1	1	3
American Indian	1	1	3
ACR total	5.51 ± 1.69		5.53 ± 1.72
Anti-dsDNA Ab (%)	33.3		22.6
Anti-Sm Ab (%)	28.9		12.5
Anti-Ro (SSA) Ab (%)	42.2		35.5
Anti-La (SSB) Ab (%)	8.9		22.6
Anticardiolipin Ab (%)	48.8		35.7
Lupus anticoagulant (%)	15.6		11.7
Anti-B2 glycoprotein Ab (%)	11.1		14.5
APS (%)	37.8		14.8
Decreased C3 (%)	8.9		4.8
Decreased C4 (%)	42.2		30.7
SLEDAI	6.02 ± 4.3		4.75 ± 4.08
BILAG	6.93 ± 5.34		5.59 ± 3.73

Ab, antibody; ACR total, the number of American College of Rheumatology 1982 revised criteria for classification of systemic lupus erythematosus; APS, antiphospholipid syndrome; BILAG, British Isles Lupus Assessment Group; F, female; M, male; SLEDAI, Systemic Lupus Erythematosus Disease Activity Index.

Prior to participation, all subjects gave informed consent to donate their blood samples and de-identified clinical information for research, and the study was approved by the Institutional Review Boards of Oklahoma Medical Research Foundation and of Temple University.

### Measurement of plasma Gas6 concentrations

Gas6 was measured with a sandwich ELISA modified from a previously developed and validated protocol [28]. Briefly, 96-well plates were coated overnight with anti-Gas6 capture antibody (goat polyclonal affinity purified IgG, R&D Systems, Minneapolis, MI, USA). The antigen was detected by a secondary biotin-conjugated antibody (Biotinylated anti-human Gas6 antibody, R&D Systems, Minneapolis, MI, USA), and a streptavidin-peroxidase conjugate (R&D Systems, Minneapolis, MI, USA) and TMB (3,3',5,5'-tetramethylbenzidine, R&D Systems, Minneapolis, MI, USA). The reaction was stopped with 2N sulphuric acid and absorbance detected at 450 nm. The absorbance at 450 nm was read with a reference wavelength set at 570 nm using a Versamax microplate reader (Molecular Devices, Sunnyvale, CA, USA). The optical density (OD) for each point was determined from the average of duplicate samples. Gas6 concentrations were determined using Softmax software (Molecular Devices, Sunnyvale, CA, USA) by applying a four-parameter logistic regression to the calibration curve prepared from duplicate serial dilutions of purified Gas6 protein (R&D Systems, Minneapolis, MI, USA). The intra-assay and inter-assay coefficient of variation (CV) were 4.52% and 11.8%, respectively.

### Measurement of plasma free protein S concentrations

Free protein S levels were quantified using the free protein S ELISA kit (Diagnostica Stago, Parsippanny, NJ, USA) according to the manufacturer's instructions. The ELISA utilizes two monoclonal antibodies, each specific for free protein S epitopes [29]. Briefly, heparinized plasma samples were diluted 1:20 in 1% BSA and duplicate 200 μl samples applied to the precoated 96-well plate. Serial dilutions of purified protein S (Hematologic Technologies Inc., Essex Junction, VT, USA) starting at 20 μg/ml were used to construct a standard curve. These were further diluted 1:20 (in 1% BSA) before being applied to the plate in duplicate. Four blank wells received 200 μl 1% BSA. The horseradish peroxidase (HRP)-conjugated secondary antibody (50 μl/well) was added immediately. The plate was developed with 200 μl/well of TMB substrate for five minutes as described for the Gas6 ELISA. The intra-assay and inter-assay CVs were 6.1% and 13.5%, respectively.

### Statistical analysis

The data were expressed as mean ± standard deviation (SD). An unpaired Student's *t*-test was used for statistical comparison of plasma Gas6 and protein S levels between matched 45 SLE patients and 45 NC and of those according to the clinical manifestations in total 107 patients with SLE. When the data did not show Gaussian distribution, the Mann-Whitney U-test was used. To detect correlation between continuous data, the Pearson correlation coefficient was applied. Prizm software (GraphPad Software, La Jolla, CA, USA) was employed for all analyses. For all tests, a *P *value of less than 0.05 was regarded as significant.

## Results

### Gas6 and free protein S concentration in SLE and NC

**Figure 1:**
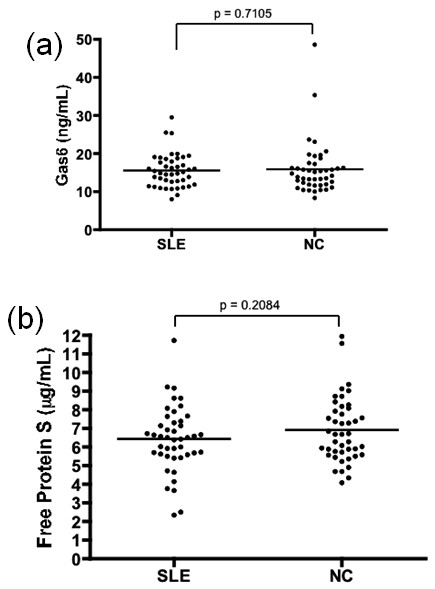
**Plasma levels of (a) Gas6 and (b) free protein S in age, gender and ethnicity matched SLE and NC**. NC, normal controls; SLE, systemic lupus erythematosus.

**Figure 2:**
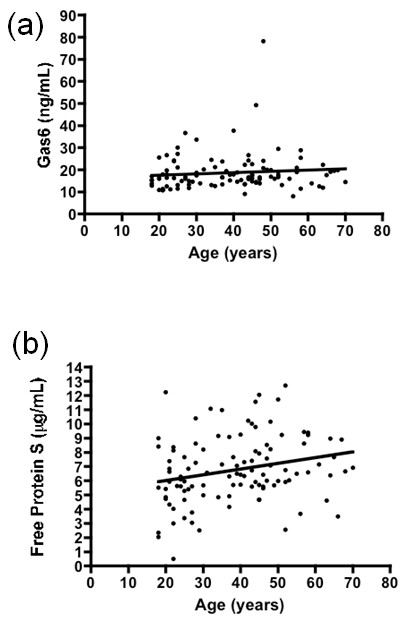
**Plasma concentrations of (a) Gas6 and (b) free protein S in SLE patients according to age**. SLE, systemic lupus erythematosus.

The plasma concentrations of Gas6 were almost identical between 45 SLE patients and age, gender and ethnicity matched 45 NC (15.55 ± 4.39 vs. 15.89 ± 6.88 ng/mL, respectively; Figure 1a). Also, there was no difference in the level of free protein S between them (6.44 ± 1.75 vs. 6.91 ± 1.74 μg/mL, respectively; Figure 1b). In examining the levels of free protein S in all 107 SLE patients, free protein S was positively correlated with age (r = 0.2405, *P *= 0.0126), but Gas6 levels did not increase with age (Figure 2). The concentrations of Gas6 and free protein S were slightly higher in females than in males, but the difference was not significant.

As Gas6 and protein S are closely related, and both can function as intermediaries for TAM receptor kinase binding to apoptotic cells, we evaluated whether their levels would be related to each other; however, there was no correlation between the concentrations of Gas6 and free protein S in SLE patient plasma (data not shown).

### Clinical characteristics and Gas6 and free protein S in SLE patients

**Figure 3:**
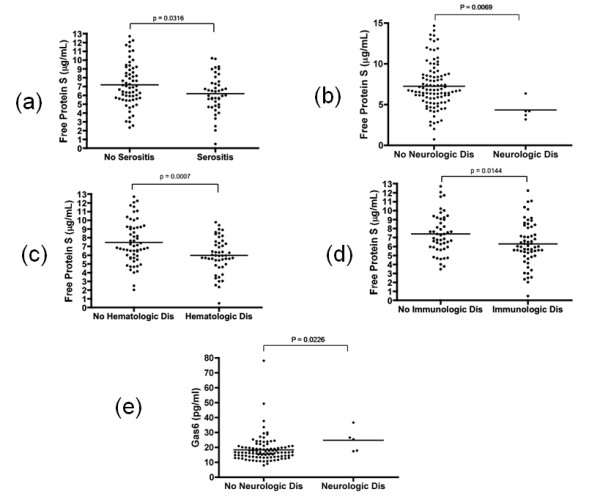
**Gas6 and free protein S levels according to the clinical manifestations in SLE**. Free protein S in patients with **(a) **serositis, **(b) **neurologic disorder, **(c) **hematologic disorder, **(d) **immunologic disorder. Gas6 plasma levels in patients with **(e) **neurologic disorder. SLE, systemic lupus erythematosus.

The concentrations of free protein S were significantly lower in SLE patients with a history of serositis, neurologic disorder, hematologic disorder, and immunologic disorder (defined by meeting 1982 revised ACR criteria than in those patients without these SLE features (Figure 3). In the patients with antiphospholipid syndrome (APS), free protein S levels were not different from patients without a history of APS. Also, free protein S in patients known to have a history of pathologic thrombosis (with or without meeting autoantibody requirements for APS) did not differ from those without thrombotic history. There was no difference in the levels of Gas6 in any subset of patients excepting neurologic disorder. Although the number of patients with a history of neurologic disorder was only five, they had elevated Gas6 levels compared with patients without a history of neurologic disorder (Figure 3e).

**Figure 4:**
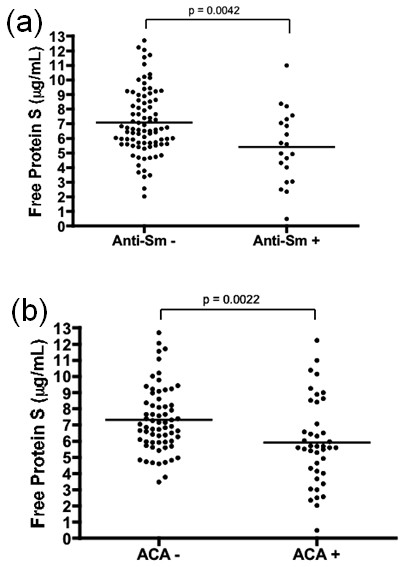
**Levels of free protein S according to the presence of autoantibodies in SLE**. Free protein S in patients with **(a) **anti-Sm and **(b)** anticardiolipin (ACA). SLE, systemic lupus erythematosus.

Free protein S was slightly lower in the patients with anti-dsDNA than those without, but the difference was not significant. The SLE patients with anti-Sm showed lower free protein S levels than those without (Figure 4a).

There are conflicting reports about free protein S levels in the patients with antiphospholipid antibody [22,23,30-33]. Our study found concentrations of free protein S to be lower in the patients with ACA (Figure 4b). However, there were no differences in the levels of free protein S between patients with and without LAC and anti-β2 glycoprotein I, respectively.

Among five SLE patients with anti-protein S antibodies, four had a history of thrombosis and three patients were positive for ACA; however, their acute levels of free protein S were not different from the patients without anti-protein S antibodies (data not shown).

### Protein S levels correlate with C3 and C4 in SLE patients

**Figure 5:**
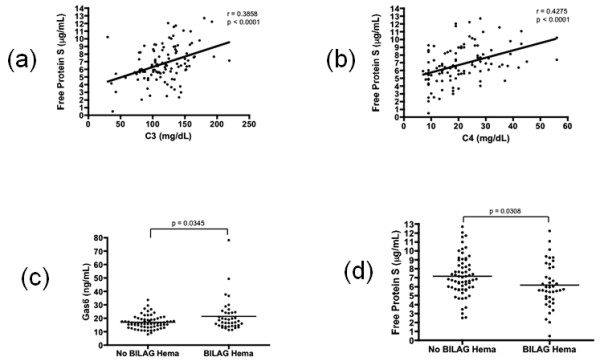
**Gas6 and free protein S levels and disease activity**. Correlation between free protein S and **(a) **C3 or **(b) **C4. **(c) **Gas 6 and **(d) **free protein S levels in patients with BILAG hematology. BILAG, British Isles Lupus Assessment Group.

The concentrations of free protein S were lower in patients with decreased C3 or C4, markers commonly used in assessing disease activity. It was striking that free protein S was positively correlated with C3 (r = 0.3858, *P *< 0.0001; Figure 5a) and C4 (r = 0.4275, *P *< 0.0001; Figure 5b).

### Disease activity and Gas6 and protein S in SLE patients

We assessed overall disease activity with SLEDAI and BILAG composite scores, but did not find any correlation in a cross-sectional population comparison with levels of Gas6 or free protein S.

In the subset of patients with active BILAG hematologic involvement, whose BILAG score is not zero, the levels of Gas6 were higher (23.05 ± 24.88 vs 16.99 ± 6.93 ng/mL, *P *= 0.008; Figure 5c) and those of free protein S were lower (6.14 ± 0.46 vs 7.16 ± 2.37 μg/mL, *P *= 0.036; Figure 5d) compared with patients without BILAG hematologic involvement. In addition, the patients with a BILAG score greater than or equal to three showed further increased concentration of Gas6 (29.93 ± 24.88 ng/ml) and those of free protein S (5.57 ± 0.46 μg/mL).

## Discussion

Abnormal clearance of apoptotic cells may be important in the development of autoantibodies in SLE. As the TAM kinases may be important in the disposition of apoptotic cells, we evaluated plasma concentrations of their ligands Gas6 and free protein S. Although the levels of Gas6 and free protein S were not different overall between patients with SLE and matched healthy controls, free protein S was decreased in subsets of SLE patients with a history of serositis, neurologic, hematologic, and immunologic disorder. It was especially noteworthy that the concentrations of free protein S were correlated with C3 and C4. Protein S was decreased in SLE patients with active hematologic disease as defined by the BILAG index. In contrast to the findings for protein S, reduced levels of Gas6 were not associated with more active disease, with the possible exception of neurologic disorder, although this analysis was limited by a small number of patients. Surprisingly, active hematologic disease as defined by BILAG revealed an unexpected association with elevated, not reduced Gas6 levels.

Protein S is a vitamin K-dependent plasma anticoagulant protein and its deficiency leads to hypercoagulability syndromes with increased risk for venous thrombosis. However, there have been limited reports about functional effects of protein S independent of its anticoagulant function. After identification of TAM kinases as receptors for protein S, this protein was shown to be required for the efficient uptake of apoptotic cells by macrophages *in vitro *[34], suggesting an important role in immune clearance. Protein S may play a particularly significant role in the removal of apoptotic cells because of its high plasma concentration, despite its apparent lower affinity for the receptor than Gas6. In the study of c-mer-mediated phagocytosis of apoptotic cells, protein S stimulated phagocytosis as well as or better than Gas6 [35,36]. Therefore, it is possible that insufficient levels of protein S may lead to inefficient clearance of apoptotic cells, resulting in exposure of cellular contents to immune cells and promoting an autoimmune response.

Several reports have suggested that the levels of free protein S may be lower in patients with SLE [22,23,32]. In the present study, there was no significant difference overall in circulating free protein S between patients with SLE and matched healthy controls. However, the concentrations of free protein S did appear to be decreased in subsets of those patients with a history of certain clinical manifestations, and low protein S correlated with acute evidence of hematologic disease activity and complement consumption. These findings support the possibility of a novel functional link between the coagulation system and distinct inflammatory responses in SLE. It is well known that there is increased cardiovascular mortality and morbidity among SLE patients, which is not fully explained by traditional risk factors [37,38]. Our results raise the possibility that, in a definable subset of patients with SLE, disease activity may lead to a decrease in the level of free protein S, which then may increase thrombogenicity. It should be considered that the protein that regulates levels of free protein S is the C4b-binding protein, which is a critical complement regulator as well [39]. The failure in our series to find decreased levels of protein S in patients with previous thrombosis could reflect the very small number of patients in that category, along with the multiple risk factors that are probably involved in the pathologic hypercoagulability of SLE. Additionally, this was a cross-sectional analysis, whereas at least one report has suggested that decreased free protein S may be more likely to be observed closer in time to a thrombotic event in patients with SLE [40]. Although decreased protein S levels may be secondary to SLE activity, we favor the hypothesis that a decrease in protein S may actually contribute to SLE pathogenesis, as discussed above and suggested by Rothlin and colleagues [41].

Previous reports have observed an association between reduced levels of free protein S and antiphospholipid antibody in SLE [23,30]. It has been suggested that acquired protein S deficiency could contribute to increased risk of thrombosis in patients with antiphospholipid antibody. However, other investigations have not confirmed an association [22,31-33]. These reports evaluated free protein S in a relatively small number of SLE patients (30 to 50 patients). In the present study assessing 107 SLE patients, free protein S levels were significantly lower only in those patients with ACA, but not in those with LAC and anti-β2 glycoprotein I. Autoantibodies directed against protein S have been associated with thrombosis in patients with APS and SLE [32,42-44]. However, the presence of anti-protein S antibodies in patients have not been found to reduce the concentrations of free protein S [32,42]. Our findings were consistent with these results although the prevalence of anti-protein S was lower in our patients (5%) than previous reports (26 to 31%).

Gas6 is a cell survival, proliferation and chemotactic factor and also a recognition bridge between phagocytes and apoptotic cells. Gas6 is present at a low concentration in plasma; however, it can be released by endothelial cells and leukocytes during serum starvation or under inflammatory conditions [19,21,45-47]. The receptors that bind Gas6 (Tyro3, Axl, and c-mer) have an immunoregulatory role, modulating macrophage activation following an initial immune stimulus [9,48]. Gas6 may thus be supposed to participate in inflammation by interfering with macrophage-lymphocyte crosstalk. Furthermore, Gas6 might be involved in other chronic systemic autoimmune diseases, such as rheumatoid arthritis and chronic inflammatory demyelinating polyneuropathy [49,50]. It has been suggested that Gas6 is involved in macrophage activation in chronic autoimmunity as an autocrine or paracrine regulatory molecule for monocytes [51].

In the present study, plasma Gas6 levels in patients with SLE were the same as in matched HC and levels were unrelated to age and gender. The concentration of Gas6 was increased in the patients with a history of neurologic disorder and acute activity in the BILAG hematology system. The latter results may reflect the inducible nature of Gas6. Basal levels of Gas6 were low, yet it is known to be upregulated in certain states of intense inflammation such as septic shock and severe acute pancreatitis [19-21]. A recent report finding that almost all Gas6 present in healthy subjects is bound by soluble Axl may explain why there is actually little free Gas6 present in either normal or SLE serum, although the extent to which our ELISA can detect axl-bound Gas6 has not been tested [52].

In SLE, free protein S was decreased in patients characterized by a history of serositis, neurologic, hematologic, and immunologic disorder. Protein S was also decreased in patients with low C3 and C4 and active hematologic activity. Thus, free protein S may be useful as a biomarker of clinical phenotype and disease activity.

Furthermore, the decrease of protein S and increase of Gas6 in patients with acute activity in the BILAG hematologic system suggests the possibility of a unique link between inflammation and thrombotic risk that could be explored mechanistically.

## Conclusions

The TAM ligands are important apoptotic debris receptors and regulators of innate immunity. Our study shows that low levels of one TAM ligand, protein S, correlate with C3 and C4 levels and with clinical manifestations of SLE. In contrast, circulating levels of Gas6, the other principal TAM ligand, have little apparent relation to SLE laboratory or clinical manifestations. These data support the view that ligation of the TAM ligands through protein S but not Gas6 is important in clearance of debris and regulation of the innate immune system in patients with SLE.

## Abbreviations

ACA: anticardiolipin; ACR: American College of Rheumatology; APS: antiphospholipid syndrome; BILAG: British Isles Lupus Assessment Group Instrument; BSA: bovine serum albumin; CV: coefficient of variation; ELISA: enzyme linked immunosorbent assay; Gas6: growth arrest-specific 6; HRP: horseradish peroxidase; Ig: immunoglobulin; NC: normal controls; SLE: systemic lupus erythematosus; SLEDAI: systemic lupus erythematosus disease activity index; TAM kinases: tyro 3, axl, mer.

## Competing interests

The authors declare that they have no competing interests.

## Authors' contributions

CHS designed and executed experiments, interpreted data, and wrote the manuscript. BH performed experiments and interpreted data. SL performed pilot experiments and interpreted data. JTM supplied samples and clinical data, interpreted results, and edited the manuscript. PLC designed experiments, interpreted data, and edited the manuscript.
